# Introduction of a novel 18S rDNA gene arrangement along with distinct ITS region in the saline water microalga Dunaliella

**DOI:** 10.1186/1746-1448-6-4

**Published:** 2010-04-08

**Authors:** Mohammad A Hejazi, Abolfazl Barzegari, Nahid Hosseinzadeh Gharajeh, Mohammad S Hejazi

**Affiliations:** 1 Branch for the Northwest and West Region, Agricultural Biotechnology Research Institute of Iran (ABRII), Tabriz, I. R. Iran; 2 Pharmacy faculty, Tabriz University, Tabriz, I. R. Iran

## Abstract

Comparison of 18S rDNA gene sequences is a very promising method for identification and classification of living organisms. Molecular identification and discrimination of different Dunaliella species were carried out based on the size of 18S rDNA gene and, number and position of introns in the gene. Three types of 18S rDNA structure have already been reported: the gene with a size of ~1770 bp lacking any intron, with a size of ~2170 bp consisting one intron near 5' terminus, and with a size of ~2570 bp harbouring two introns near 5' and 3' termini. Hereby, we report a new 18S rDNA gene arrangement in terms of intron localization and nucleotide sequence in a Dunaliella isolated from Iranian salt lakes (ABRIINW-M1/2). PCR amplification with genus-specific primers resulted in production of a ~2170 bp DNA band, which is similar to that of *D. salina *18S rDNA gene containing only one intron near 5' terminus. Whilst, sequence composition of the gene revealed the lack of any intron near 5' terminus in our isolate. Furthermore, another alteration was observed due to the presence of a 440 bp DNA fragment near 3' terminus. Accordingly, 18S rDNA gene of the isolate is clearly different from those of *D. salina *and any other Dunaliella species reported so far. Moreover, analysis of ITS region sequence showed the diversity of this region compared to the previously reported species. 18S rDNA and ITS sequences of our isolate were submitted with accesion numbers of EU678868 and EU927373 in NCBI database, respectively. The optimum growth rate of this isolate occured at the salinity level of 1 M NaCl. The maximum carotenoid content under stress condition of intense light (400 μmol photon m^-2 ^s^-1^), high salinity (4 M NaCl) and deficiency of nitrate and phosphate nutritions reached to 240 ng/cell after 15 days.

## Background

Dunaliella is an unicellular halotolerant microalga with a great potential as transgenic bioreactor [1], and more significantly, food source and pharmaceuticals due to its ability to accumulate large amounts of carotenoids [2]. Isolation and identification of novel species and strains from natural habitats is the main purpose in the path of obtaining superior productive strains. On one hand due to environmental adaptation and lacking of a cell wall, a certain Dunaliella isolate may exhibit different morphological and physiological behaviour in different conditions [3]. On the other hand, wide geographic distribution of saline systems and required distinctive adaptations to these environments lead to extensive diversity of the organisms living in these systems [4]. In addition, slight molecular-based phylogenetic differences of Dunaliella species can reveal deep distinction in production of metabolites such as carotenoids [5]. Hence, molecular characterization provides an important tool for exploring biodiversity of Dunaliella and better understanding of its taxonomy.

Identification and classification of the organisms based on conserved and variable regions of 16S or 18S rDNA is a common procedure in taxonomy studies [6]. 18S rDNA gene has been used for molecular identification of different species of Dunaliella as eukaryotic microorganism [7-9]. Besides using intron sizing method, particular 18S rDNA fingerprint profiles were reported as an indicator for hyperproducer species [10]. Ribosomal spacer sequences, including ITS regions have been also frequently utilized for discrimination of genetic variation in green algae [11-15]. Sequence comparison of the ITS region was utilized to predict genetic relatedness and to study phylogeny and taxonomy of Dunaliella [5,15].

18S rDNA gene in Dunaliella genus contains relatively conserved region of exon(s) and variable region of intron(s). It is worth nothing that some species don't have any intron. Wilcox *et al*. (1992) declared that the type of introns present in the genus Dunaliella belongs to group I [16]. Different secondary structure of group I introns makes it distinguished from those of group II and III introns [17]. Group I introns display widespread but irregular distribution in the organisms [18]. These introns are frequently present in lower eukaryotes, especially algae and fungi [19,20]. Since nuclear rDNAs are heritable, group I introns can be used as phylogenetic markers [20]. Olmos *et al*. (2000 and 2002) [7,8] designed and used conserved primers (directed to exon region of 18S rDNA) and species-specific primers (directed to the introns of 18S rDNA) to identify some species. Based on these studies, some intron arrangements in 18S rDNA region were identified in different species of Dunaliella. They reported that 18S rDNA gene of *D. tertiolecta *(~1770 bp) lacks any intron, 18S rDNA gene of *D. salina *(~2170 bp) has just one intron after the first exon at 5' terminus that we call as intron 1. 18S rDNA gene in both species *D. parva *and *D. bardawil *(~2570 bp) possess two introns; one after the first exon at 5' terminus and the other after the second exon at 3' terminus that we name them as introns 1 and 2, respectively. More recently partial sequence of *D. viridis *18S rDNA gene (GenBank: DQ009776) has been submitted with a size of 2494 bp in NCBI [21]. *D. viridis *has a longer intron compared to the introns 1 and 2, after the first and before the second exon, again at 5' terminus. We name this intron as intron 3. As molecular aspects of these microorganisms have not been studied enough, and so little is known about the different types of intron arrays, there is possibility of revealing diverse structure of 18S rDNA region. This may lead to discovery of new species/subspecies. This research was conducted to study the structure of 18S rDNA gene in one of the Iranian isolates of Dunalilla. The investigation was accompanied by studying ITS region simultaneously. 18S rDNA and ITS region of this isolate were submitted at NCBI by accession numbers of EU678868 and EU927373, respectively.

## Results and Discussion

### PCR Amplification of 18S rDNA gene

**Figure 1:**
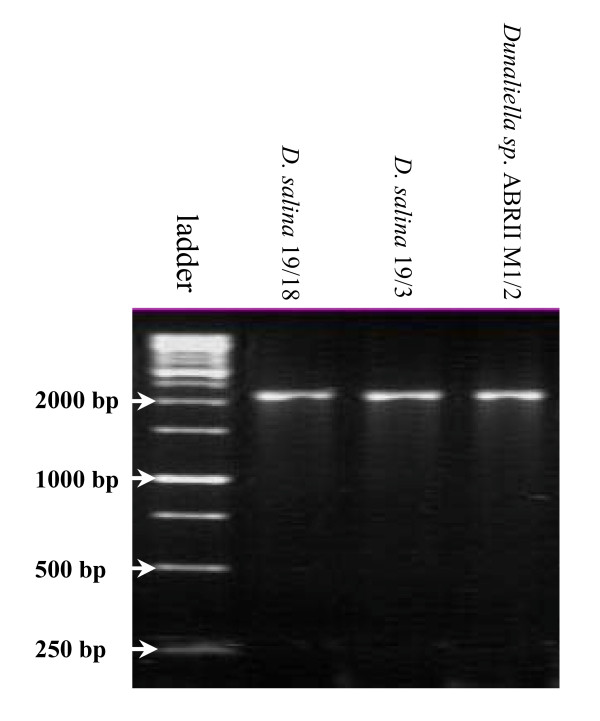
**Amplification with conserved primers of MA1-MA2**. The PCR product was analyzed by electrophoresis using 1% agarose gel and marked using 1 kb DNA ladder (Fermentas Co.).

DNA extraction was performed when the cell density of liquid culture was approximately 8 × 10^3 ^cell/ml. In order to confirm the genus of the isolate, PCR amplification of 18S rDNA gene was performed using MA1-MA2 primers. These primers allow amplification of almost full length of 18S rDNA in different Dunaliella species. In addition, to investigate the species level of our isolate, PCR amplification was carried out using the species-specific primers. PCR amplification with MA1-MA2, resulted in production of a ~2170 bp DNA band (Figure 1). The amplification with species-specific primers of DSs-MA2, DPs-MA2 and DBs-MA2 created no DNA fragment.

Using MA1-MA2, the presence of the DNA amplicon confirmed the genus of our isolate as Dunaliella. Concerning the species of Dunaliella, it is reminded that *D. tertiolecta*, *D. salina*, *D. parva *and *D. bardawil *produce ~1770, ~2170, ~2570 and ~2570 bp DNA fragments with these primers, respectively [7,8]. Based on the submitted sequence of *D. viridis *(GenBank: DQ009776), 18S rDNA band size of about 2500 bp is expected using these primers. As shown in figure 1, the size of the amplified DNA was similar to 18S rDNA size of two 19/3 and 19/18 strains of *D. salina*.

Moreover, according to Olmos *et al*. (2000 & 2002) DSs-MA2 should amplify a DNA fragment of ~750 bp in *D. salina*, DPs-MA2 should produce DNA amplicon with the size of ~1050 bp in *D. parva *and DBs-MA2 should amplify a fragment of ~1000 bp in *D. bardawil*. Because *D. tertiolecta *lacks any intron, no DNA amplicon is expected with species-specific primers. Taken these results together, although the size of 18S rDNA gene was similar to that of *D. salina*, creation of no PCR product with any of species-specific primers including DSs-MA2 suggests that 18S rDNA profile in our isolate is different from the others studied so far.

### RFLP and Sequence analysis of 18S rDNA gene

The accuracy of the discrepancy from *D. salina *was approved by RFLP analysis. The restriction patterns of 18S rDNA gene of the isolate ABRIINW-M1/2 exhibited different polymorphism (data not shown) comparing with both strains of *D. salina *19/3 and 19/18. When digested with Taq I, 18S rDNA of our isolate was cut into 5 restricted fragments, while in both standard *D. salina *samples 4 different fragments were produced.

**Figure 2:**
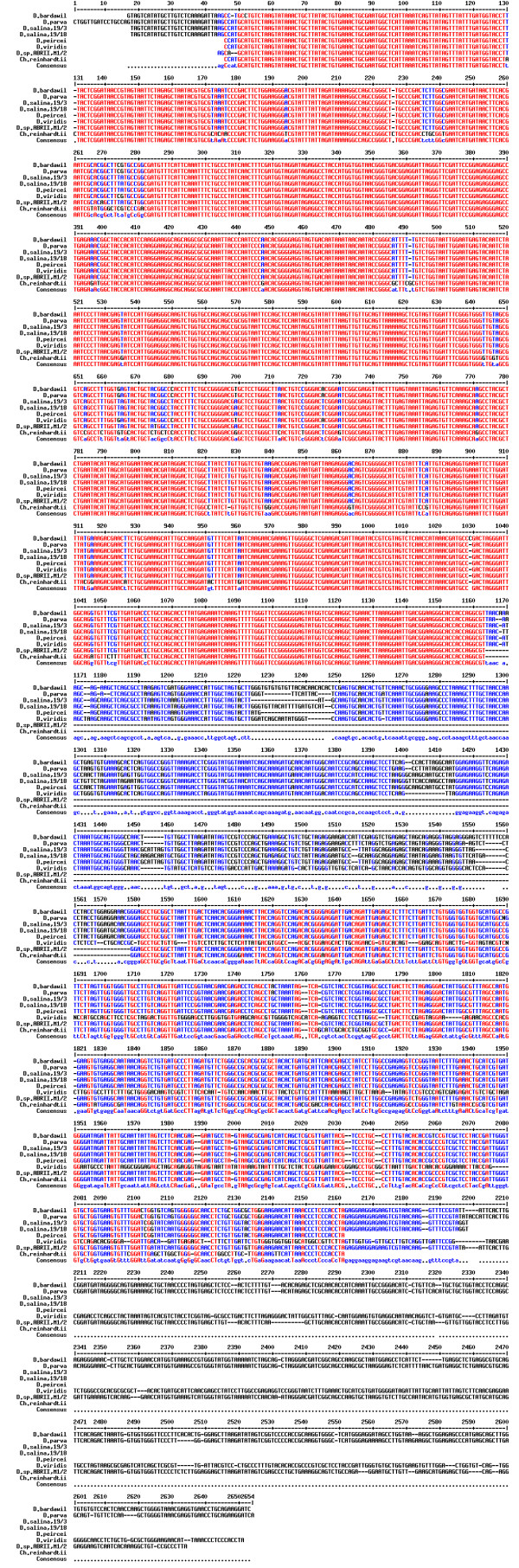
**18S rDNA sequence alignment of *Dunaliella sp***. ABRIINW-M1/2 and other Dunaliella species. Data for other species were gathered from NCBI. The conserved regions of the gene are demonstrated in different colour (red).

18S rDNA sequencing was performed in order to obtain detailed information regarding the structure of this region in our isolate. The sequence of our isolate along with those of other Dunaliella species were aligned multiply (Figure 2). Due to the lack of any intron in *D. tertiolecta *18S rDNA gene, this species was excluded from the figure and further analysis. As seen in figure 2, our isolate (ABRIINW-M1/2) lacks a fragment composed of 414 nucleotides extended from 1164 to 1578 corresponding to the intron 1. Moreover, the gene of our isolate possesses a variable region of 440 nucleotides at 3' terminus starting from about nucleotide 2195. Numbering was conducted according to the 18S rDNA sequence of *D. parva *with accession number of M62998. Lack of intron 1 and presence of intron 2 in our isolate discriminate the isolate from *D. salina *which contains only intron 1, as we already discussed in the introduction. It should be noted that according to DNA alignment, partially submitted 18S rDNA sequence of *D. peircei *(strain UTEX LB 2192, GenBank: DQ009778) whose fingerprint profile was unpublished includes a corresponding region extending from about 1164 to 1580, in the same position as the intron of *D. salina*.

*D. parva *and *D. bardawil *contain both intron 1 and intron 2 with a 18S rDNA length of about 2570 nucleotides. This is while, our isolate lacks intron 1 and harbours a 18S rDNA with a size of about 2170 nucleotides. Therefore, the isolate would be different from *D. parva *and *D. bardawil*.

**Figure 3:**
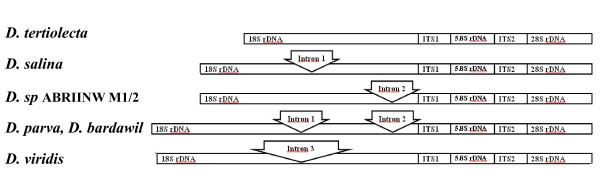
**Diagram showing the position of intron insertions in 18S rDNA gene of Dunaliella species**. 18S rDNA arrangement is demonstrated in 5 group based on the insertion site of introns. The names of the species wich have the corresponding type of arrangement is written in left side.

*D. viridis *contains only one longer intron compared with introns 1 and 2. It is settled after the first and before the second exon in a position near to 5' terminus with a size of about 810 nucleotides. Accordingly, the isolate does not belong to the species of *D. viridis*, either. The position and arrangement of introns in different forms is depicted in Figure 3.

18S rDNA sequence of our isolate was submitted at NCBI by the name of *Dunaliella sp*. ABRIINW-M1/2 and accesion number of EU678868. ABRIINW stands for Agricultural Biotechnology Institute of Iran, Northwest and West region where this research was performed in.

**Figure 4:**
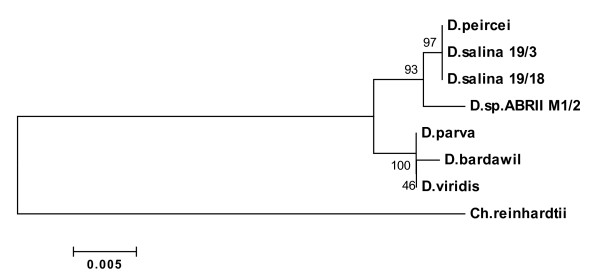
**Phylogram based on 18S rDNA gene for the species of Dunaliella and *Dunaliella sp***. ABRIINW M1/2. The tree is constructed using neighbor-joining method. Bootstrap values were calculated from 1000 replicates.

All cluster analyses using Neighbor Joining (NJ), Unweighted Pair Group Method with Arithmatic Mean (UPGMA), Maximum Parsimony (MP) and Minimum Evolution (ME) of MEGA4 showed similar clade arrangement. They strongly supported (88-93% bootstrap values) the branch including *Dunaliella sp*. ABRIINW-M1/2 differing from other taxa. Figure 4 shows the result of bootstrap analysis of NJ tree. All sequences in the dendrogram were divided into three lineages of A, B and C. As seen in the figure, *Dunaliella sp*. ABRIINW-M1/2 appeared as individual entity (clade B) differing from two strains (19/3 and 19/18) of *D. salina *and *D. peircei *(clade A) with high bootstrap value (93%). The cluster including clades A and B was divergent from clade C which is composed of *D. viridis*, *D. bardawil *and *D. parva*.

**Figure 5:**
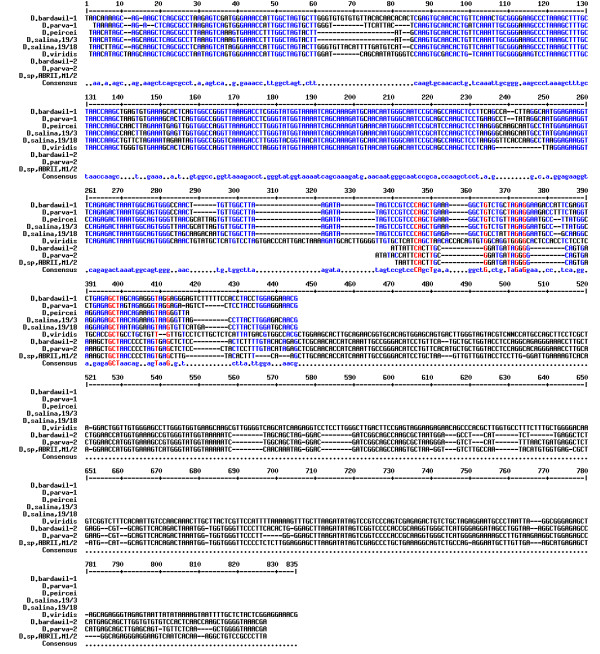
**18S rDNA intron alignment of *Dunaliella sp***. ABRIINW-M1/2 and other Dunaliella species. Data for other species were gathered from NCBI. The conserved regions of the gene are demonstrated in different colour (red).

In the next step, the intron location of various Dunaliella species were marked and they were aligned seperately (Figure 5). This analysis showed that the intron of *Dunaliella sp*. ABRIINW-M1/2, displayed the highest similarity of 79% and 83% with intron 2 present in *D. parva *and *D. bardawil*.

**Figure 6:**
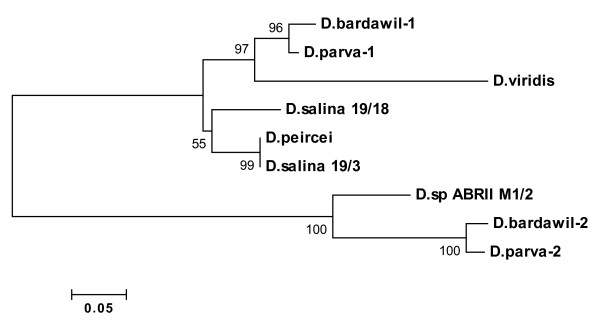
**Phylogram based on the intron of 18S rDNA gene for the species of Dunaliella and *Dunaliella sp***. ABRIINW M1/2. The first and second introns of the species *D. parva *and *D. bardawil *are represented with 1 and 2, respectively. The tree is constructed using neighbor-joining method. Bootstrap values were calculated from 1000.

In order to better investigate the similarity of intron regions, phylogenetic tree was calculated using neighbor-joining (NJ). The result of bootstrap analysis of NJ tree is displayed in Figure 6. The intron sequences were clustered in two separate groupings. The intron of *D. salina *19/3 and *D. salina *19/18 together with the corresponding region in *D. peircei *showed homology with the clade containing intron 1 of *D. parva *and *D. bardawil *which has lineage with *D. viridis*. The single intron of ABRIINW-M1/2 was grouped with intron 2 of *D. bardawil *and *D. parva*. These results further approved the novelty of the gene arrangement.

### PCR amplification and sequence analysis of ITS region

**Figure 7:**
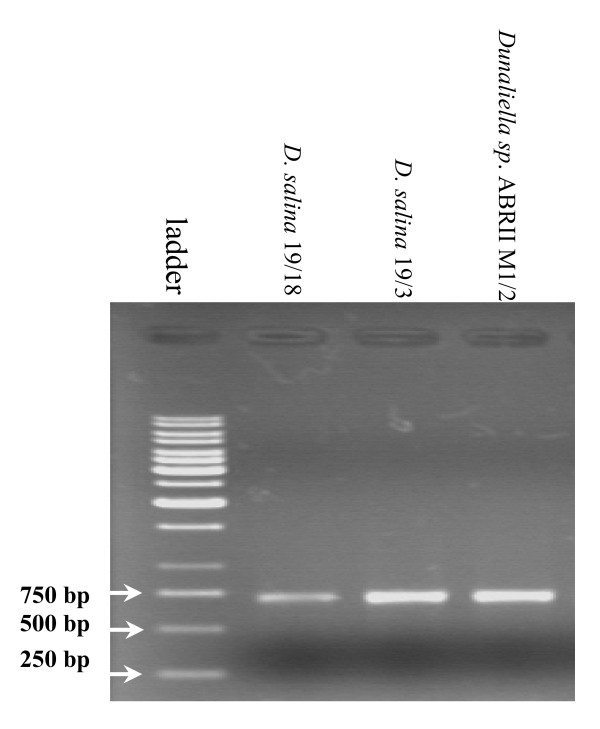
**ITS amplification with the primers of AB1-AB2**. The PCR product was analyzed by electrophoresis using 1% agarose gel and marked using 1 kb DNA ladder (Fermentas Co.).

**Figure 8:**
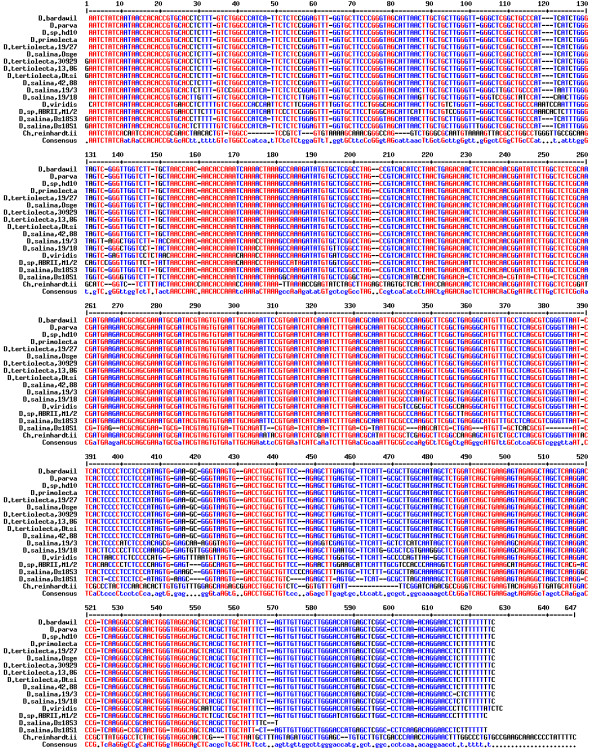
**ITS (ITS1, 5.8 rDNA and ITS2) sequence alignment of *Dunaliella sp***. ABRIINW-M1/2 and other Dunaliella species. Data for other species were gathered from NCBI.

In addition to 18S rDNA gene, we PCR ampified and sequenced Internal Transcribed Sequence (including ITS1, 5.8 rDNA and ITS2) of our isolate to assess the diversity with other members of Dunaliella genus. PCR amplification of ITS region resulted in production of a single band with a size of ~700 bp (Figure 7) similar to its size in the other species. This length was expected as it was found that ITS region in Dunaliella does not show the length variation at intra- or interspecific level (10). Then the sequence was aligned with 16 different strains (table 1) whose ITS sequences were fully recorded at NCBI including the outgroup of *Ch. reinhardtii *(Figure 8). ITS nucleotide sequence of *Dunaliella sp*. ABRIINW-M1/2 exhibited various similarities ranging from 78-88% with others. It showed the highest and lowest similarity with *D. viridis *and *D. salina *Ds18S3, respectively. Since ITS sequence of *D. peircei *was submitted as separate sets of ITS1 and ITS2 and they didn't show any significant similarity with the corresponding regions of the others, it was excluded from the analysis. The ITS sequence of *Dunaliella sp*. ABRIINW-M1/2 was registered with accession number of EU927373 in NCBI database.

**Table 1:** List of Dunaliella strains investigated in this study

Dunaliella strains	Accession NO. (18S rDNA)	Accession NO. (ITS region)
*Dunaliella sp*. ABRIINW-M1/2	EU678868	EU927373
*Dunaliella bardawil*	AF150905	DQ116744
*Dunaliella parva*	M62998	DQ116746
*Dunaliella salina *strain CCAP 19/3	EF473743	EF473744
*Dunaliella salina *strain CCAP 19/18	EF473745	EF473746
*Dunaliella viridis *strain CONC002	DQ009776	DQ377098
*Dunaliella peircei *strain UTEX LB 2192	DQ009778	
*Dunaliella salina *SAG 42.88		EF473741
*Dunaliella salina *Ds18S3		FJ360758
*Dunaliella salina *Ds18S1		FJ360756
*Dunaliella salina *Dsge		EF473732
*Dunaliella tertiolecta *CCAP 19/27		EF473748
*Dunaliella tertiolecta *ATCC 30929		EF473742
*Dunaliella tertiolecta *SAG 13.86		EF473738
*Dunaliella tertiolecta *Dtsi		EF473730
*Dunaliella primolecta*		DQ116745
*Dunaliella sp*. hd10		DQ116747
*Chlamydomonas reinhardtii*	AB511836	AB511842

**Figure 9:**
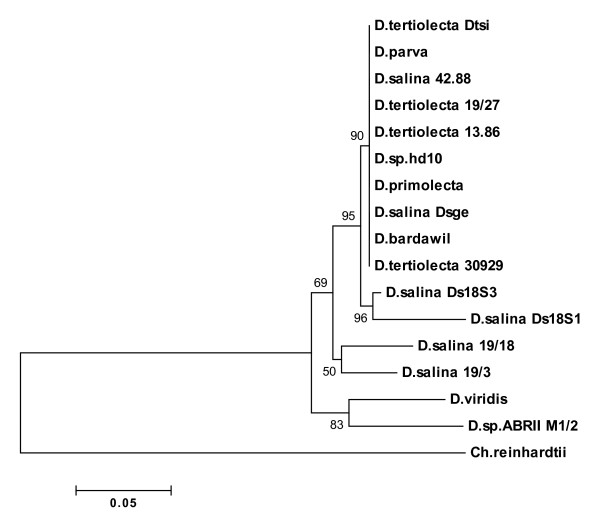
**Dendrogram showing the relationship among *Dunaliella sp***. ABRIINW-M1/2 and species of Dunaliella. The tree is based on ITS region and was constructed using the neighbor-joining method. Bootstrap values were calculated from 1000.

In order to study phylogenetic relationship between our isolate and the other Dunaliella species based on ITS region, different algorithms available at MEGA4 were utilized. Figure 9 shows the corresponding dendrogram established by NJ. As expected the outgroup, *Ch. reinhardtii *was the divergent from other taxa related to Dunaliella species. The cluster analysis of 16 taxa including our isolate demonstrated their association into four clades. Clade A contains all strains of *D. tertiolecta *(CCAP 19/27, ATCC 30929, SAG 13.86 and Dtsi), two strains of *D. salina *(SAG 42.88 and Dsge), *D. primolecta*, *D. parva*, *D. bardawil *and *Dunaliella sp*. hd10. Clade B consists of two strains of *D. salina *Ds18S1 and *D. salina *Ds18S3. Clade C includes two other strains of *D. salina *(CCAP 19/3 and CCAP 19/3) with weak bootstrap value of 50%. The isolate of our interest, *Dunaliella sp*. ABRIINW-M1/2 appeared in clade D together with *D. viridis *strain CONC002 (bootstrap value: 83%). The tree supported the split between this clade (D) and all other taxa included in clades A, B and C.

### Salt tolerance and carotenoid production ability

Salt tolerance and growth experiments showed that our isolate can grow at different salinity levels ranging from 0.5 to 4 M NaCl but the highest cell concentration was obtained at 1 M NaCl (14.4 × 10^8 ^cell/ml).

The carotenoied content of the cells, cultivated under stress condition of high light intensity (400 μmol photon m^-2 ^s^-1^), high salinity (4 M NaCl) and nutrient limitation (deficiency of KNO_3 _and KH_2_PO_4_), was measured on days 12, 15 and 21. The carotenoid content of the cells reached to the maximum amount of 240 ng/cell after 15 days and remained constant till day 21.

## Conclusion

This work demonstrates a new 18S rDNA arrangement in Dunaliella genus. Although the size of 18S rDNA is similar to that of *D. salina*, position and nucleotide structure of the intron is clearly different. The isolate differs from *D. bardawil *and *D. parva *by the size of 18S rDNA and number of the introns. Likewise, it varies from *D. viridis *in terms of 18S rDNA gene length, and the size and position of the intron. Further, analysis of ITS region sequence indicated that this region in our isolate shows similarity ranging from 78-88% with those of previously known species. Based on the phylogenetic analysis, this isolate with its closest related taxon of *D. viridis *was divergent from all other taxa. Thereupon, the reported sequences are considered as novel 18S rDNA arrangement and distinct ITS region. Optimum growth of this isolate occurred at 1 M NaCl and Maximum total carotenoid was measured as 240 ng/ml. However, complementary morpho-physiological studies of the isolate are needed to elucidate if the isolate is a new species/subspecies.

## Materials and methods

### Dunaliella isolation and culture condition

Sampling was attempted from Maharlou saline lake of Iran. 10 ml of the specimens were cultivated in a liquid medium described by Hejazi and Wijffles [22] containing 9% NaCl. Dunaliella single clonies were obtained by spreading the liquid culture on the solidified medium containing 1.8% Agar. Then each single clony was cultured in 50 ml liquid medium. The culture was incubated at 26°C and irradiance of of 80 μmol photon m^-2 ^s^-1 ^in the photoperiod of 16: 8 (L: D). Two strains of 19/3 and 19/18 *D. salina *were obtained from CCAP (Culture Collection of Algae and Protozoa) as standard strains of *D. salina*.

### Genomic DNA extraction

DNA extraction was conducted according to the protocol developed in our laboratory: 1.5 microlitre of the green algae culture was centrifuged for 5 min at 5000 rpm, and supernatant was discarded. The micoalgal cells were suspended in the lysis buffer containing CTAB 10 g/l, NaCl 80 g/l, Trise 12 g/l, EDTA 7 g/l, LiCl 2 g/l, PVP 2 g/l and shaked to be well mixed. After incubation at 60°C for 10 min, the mixture was centrifuged for 5 min at 10,000 rpm. Then equal volume of Chloroform-isoamylalcohol (24:1) was added and again centrifuged for 5 min at 12000 rpm. Upperphase supernatant was transferred into a new tube and genomic DNA was precipitated by addition of equal volume of isopropanol. The tube was kept for 10 min at -20°C, then centrifuged at 13000 rpm for 5 min. Supernatant was discarded and the DNA pellete was finally washed twice using ethanol 70%.

### PCR amplification of 18S rDNA

To amplify 18S rDNA gene, two conserved primers called MA1 and MA2, corresponding to conserved regions of 5' and 3' termini, respectively, were used as forward and reverse primers [7]. PCR reactions were performed in 50 μl containing 20 ng genomic DNA in TE (Tris/EDTA) buffer, pH 8 [23] and 50 ng of the primers using 1× PCR Master Kit (CinnaGen PCR Masster Kit, Cat. No. PR8250C). Amplification was performed using 32 cycles in a TECHNE Thermal Cycler (Model: FTGRAD2D). The amplification was achieved according to the method described by Olmos *et al*. (2000).

Based on the differences in the number and sequence of introns in various Dunaliella species, Olmos *et al*. (2000 & 2002) designed three species-specific primers of DSs (5'-GCAGGAGAGCTAATAGGA-3'), DPs (5'-GTAGAGGGTAGGAGAAGT-3') and DBs (5'-GGGAGTCTTTTTCCACCT-3') for descrimination of the species. Primer DSs is directed to the single intron of *D. salina*. Primers DBs, DPs are directed to the second intron of *D. parva *and second intron of *D. bardawil*, respectively. They were used as forward primers along with the primer MA2 as reverse primer for species identification according to the method suggested by Olmos *et al*. (2002).

### PCR amplification of ITS region

To design primers for ITS region, including ITS1, 5.8S rDNA and ITS2, full ITS sequence of 6 strains namely *Dunaliella bardawil *DQ116744, *Dunaliella parva *DQ116746, *Dunaliella salina *CCAP 19/3 EF473744, *Dunaliella salina *CCAP 19/18 EF473746, *Dunaliella tertiolecta *CCAP 19/27 EF473748 and *Dunaliella viridis *CONC 002 DQ377098 were imported to Oligo5 program. Two primers of AB1 (5'-AATCTATCAATAACCACACCG-3') and AB2 (5'-TTTCATTCGCCATTACTAAGG-3') were designed according to conserved sequences flanking ITS region in Dunaliella species. These primers cover nucleotides 1-21 of ITS1 and 80-100 of 28S rDNA gene, respectively. The target sequence was amplified in total mixture volume of 50 μl in above mentioned condition. PCR amplification was carried out as follows: 5 min at 95°C as initial denaturing time, 35 cycles of 94°C for 1 min, 57°C for 50 sec and 72°C for 1 min followed by final extention step of 72°C for 10 min.

### Restriction Fragment Length Polymorphism (RFLP) of amplified 18S rDNA gene

For preliminary assessment of the sequence difference in certain isolates/species which were assumed to be correlated, the PCR products of the relevant samples were exposed to restriction endonuclease of Taq I. According to the protocol of manufacters (Germany, Fermentase), 250 ng of the product was digested with 5 U of the enzyme in 37 C for 3 hours. Restriction fragments were resolved through 2% agarose gel and were stained by ethidium bromide.

### Purification and sequencing of PCR products

PCR amplicons were purified using PCR purification kit (Roche) according to the manufacturer's instructions. Then, the purified products were sequenced by Macrogen company (Korea). Using BLAST software, the determined sequences were compared with the sequences deposited in NCBI GenBank as 18S rDNA and ITS regions of different Dunaliella species.

### Phylogenetic analysis of sequences

To analyze the phylogeny of the isolated Dunaliella, alignment of the sequences was performed with the sequences of different Dunaliella species which were submitted in NCBI database as complete sequence (table 1). Multiple alignment was performed using Expasy, Multalin [At GENOTOUL BIOINFO] website. Four algorithms available at MEGA4 ver. 4: Neighbour-Joining (NJ), Unweighted Pair Group Method with Arithmatic Mean (UPGMA), Maximum Parsimony (MP) and Minimum Evolution (ME) were employed to construct phylogenetic relationships. Using NJ, the evolutionary distances were computed using the Maximum Composite Likelihood model and reliability of the branches was assessed by bootstrapping the data with 1000 replicates. Phylogenetic studies included *Chlamydomonas reinhardtii *(GenBank: AB511836 and AB511842 for 18S rDNA and ITS region, respectively) as the outgroup.

### Salt tolerance and carotenoid production ability

To determine the optimum growth rate, the isolate of our interest was grown at five different concentrations of NaCl (0.5, 1, 2, 3 and 4 M). The flasks were maintained at 26°C and of 80 μmol photon m^-2 ^s^-1 ^in 16 h light: 8 h dark cycle. Growth behaviour of the isolate was measured every two days using a Neubaur haemocytometer.

For determination of carotenoid content, the described liquid medium was prepared with 4 M NaCl. Further, the cultures were exposed to deficiency of nitrate (KNO_3_) and phosphate (KH_2_PO_4_). The flasks were shaked and maintained at constant high light intensity of 400 μmol photon m^-2 ^s^-1^. The stress condition was considered to obtain the maximum carotenoid production. Both optimum growth salinity and carotenoid studies were conducted in triplicate samples.

Carotenoid extraction was carried out after 12, 15 and 21 days cultivation. Its concentrations were deternined spectrophotometrically according to the method descrýbed by Hejazi *et al*. [24].

## Competing interests

The authors declare that they have no competing interests.

## Authors' contributions

MAH conceived the study. MAH, AB, and NHG performed experimental work and analysis of data and wrote the manuscript. MSH participated in data analysis and writing of the manuscript. All authors read and approved the final manuscript.
